# NetTurnP – Neural Network Prediction of Beta-turns by Use of Evolutionary Information and Predicted Protein Sequence Features

**DOI:** 10.1371/journal.pone.0015079

**Published:** 2010-11-30

**Authors:** Bent Petersen, Claus Lundegaard, Thomas Nordahl Petersen

**Affiliations:** Department of Systems Biology, Center for Biological Sequence Analysis (CBS), Technical University of Denmark, Lyngby, Denmark; Griffith University, Australia

## Abstract

β-turns are the most common type of non-repetitive structures, and constitute on average 25% of the amino acids in proteins. The formation of β-turns plays an important role in protein folding, protein stability and molecular recognition processes. In this work we present the neural network method NetTurnP, for prediction of two-class β-turns and prediction of the individual β-turn types, by use of evolutionary information and predicted protein sequence features. It has been evaluated against a commonly used dataset BT426, and achieves a Matthews correlation coefficient of 0.50, which is the highest reported performance on a two-class prediction of β-turn and not-β-turn. Furthermore NetTurnP shows improved performance on some of the specific β-turn types. In the present work, neural network methods have been trained to predict β-turn or not and individual β-turn types from the primary amino acid sequence. The individual β-turn types I, I', II, II', VIII, VIa1, VIa2, VIba and IV have been predicted based on classifications by PROMOTIF, and the two-class prediction of β-turn or not is a superset comprised of all β-turn types. The performance is evaluated using a golden set of non-homologous sequences known as BT426. Our two-class prediction method achieves a performance of: MCC  = 0.50, Q_total_ = 82.1%, sensitivity  = 75.6%, PPV  = 68.8% and AUC  = 0.864. We have compared our performance to eleven other prediction methods that obtain Matthews correlation coefficients in the range of 0.17 – 0.47. For the type specific β-turn predictions, only type I and II can be predicted with reasonable Matthews correlation coefficients, where we obtain performance values of 0.36 and 0.31, respectively.

**Conclusion:**

The NetTurnP method has been implemented as a webserver, which is freely available at http://www.cbs.dtu.dk/services/NetTurnP/. NetTurnP is the only available webserver that allows submission of multiple sequences.

## Introduction

The secondary structure of a protein can be classified as local structural elements of α-helices, β-strands and coil regions. The latter is often thought of as unstructured regions, but do contain ordered local structures such as α-turns, γ-turns, δ-turns, π-turns, β-turns, bulges and random coil structures [1], [2]. Turns are defined by a distance that is less than 7 Å between Cα-atoms *i*, *i+2* for γ-turns, *i*, *i+3* for β-turns, *i*, *i+4* for α-turns and *i*, *i+5* for π-turns. Within each turn class, a further classification can be made based on the backbone dihedral angles phi and psi.

β-turn types are classified according to the dihedral angles (Φ and ψ) between amino acid residues *i+1* and *i+2* [3], [4]. The standard nomenclature for the β-turn types are: I, I', II, II', VIII, VIa1, VIa2, VIb and IV [5]. The dihedral angles for the 9 turn types are shown in Table S1.

A β-turn thus involves four amino acid residues, where the two central residues, *i+1* and *i+2*, cannot be helical. Occasionally β-turns are stabilized with a hydrogen bond between the N-H of residue *i* and the C = O of residue *i+3*. In cases where no hydrogen bond is found, the β-turn is referred to as an open β-turn [6].

β-turns are the most abundant type of turn structure found in proteins. They play an important role in the formation of compact shapes in proteins, and are often referred to as orienting structures due to the fact that they have the ability to reverse the direction of a protein chain. Approximately 25% of amino acids in protein structures are located in a β-turn and about 58% of all β-turns are composed of different overlapping β-turn types [5].

Prediction of β-turns started in the 1970s where the first β-turn prediction methods relied on statistical information derived from three-dimensional protein structures [1], [5], [7], [8], [9], [10]. The method implemented by Zhang and Chou [11] considered the pairing of the first and the fourth residue, and of the second and the third residue in a β-turn, and the predictive performance reached a Matthews correlation coefficient of 0.17. The work by Fuchs and Alix [6] used statistical methods combined with information obtained from regular secondary structure prediction. Combined with propensity scores and use of evolutionary information, they achieved a Matthews correlation coefficient of 0.41.

The most accurate β-turn predictors today utilize machine-learning methods, although the first approaches did not reach the performance obtained by the best statistical methods. The first method that predicted β-turns by use of neural networks was implemented by McGregor et al. [12] achieving a Matthews correlation coefficient of 0.20. Ten years later Shepherd et al. [13] added secondary structure predictions and the use of a two-layered network architecture (BTPRED method) and obtained a Matthews correlation coefficient of 0.35. Using a k-nearest-neighbor approach, a method by Kim [14] reached a Matthews correlation coefficient of 0.40. Kaur et al. [15], [16] further enhanced the performance of β-turn prediction by use of secondary structure predictions and evolutionary information in form of position specific scoring matrices as input to the neural networks (BetaTPred2 method) [17]. Using a uniform dataset of 426 non-homologues proteins (BT426) they obtained a Matthews correlation coefficient of 0.43. Recently support vector machines have become more widely used in the field of β-turn prediction, which is seen by the work of Zhang et al. [18] and Liu et al. (E-SSpred method) [19]. Using support vector machines with multiple alignments and secondary structure predictions from PSIPRED [20], Zhang et al. obtained a Matthews correlation coefficient of 0.45, which was slightly higher than the E-SSpred method. E-SSpred reached a Matthews correlation coefficient of 0.44, but they were the first to break the 80% accuracy (Q_total_) barrier and achieved a Q_total_ of 80.9%, compared to 77.3% by Zhang et al.

Zheng and Kurgan [21] applied support vector machines using a feature space consisting of position specific scoring matrices and secondary structure predictions from four different methods. After feature reduction, using 90 features, they obtained a Matthews correlation coefficient of 0.47. A similar performance was reached by Hu and Li [22] with a method based on support vector machines using features from position conservation scoring functions. Their method obtained a Matthews correlation coefficient of 0.47 using 7-fold cross-validation on the BT426 dataset.

β-turns are often accessible and generally hydrophilic, two characteristics of antigenic regions [1]. For this reason they are suitable candidates for being involved in molecular recognition processes. Pellequer et. al. [23] found that 50% of the linear B-cell epitopes in a small dataset of 11 proteins were located in turn regions. Thus prediction of β-turns could improve the prediction of epitopes. Krchnak et al. [24] found that the parts of a protein, which can induce protein-reactive anti-peptide anti-bodies, mostly reside in regions that have a high tendency to form β-turns. A more recent article by the same authors showed that peptide sequences including a β-turn conformation tended to induce antibodies that were able to cross-react with the parent protein [25]. β-turn and coil conformations has also previously been used to predict linear epitopes [26]. Furthermore, β-turn types I and II, are important for binding between phospho-peptides and SH2-domains [27].

NetTurnP is a new method trained to predict β-turns and the corresponding β-turn type using two layers of neural networks. An improved performance is shown compared to other prediction methods. It has been implemented as a webserver, which is freely accessible at http://www.cbs.dtu.dk/services/NetTurnP/.

## Results

### Neural network setup

**Figure 1:**
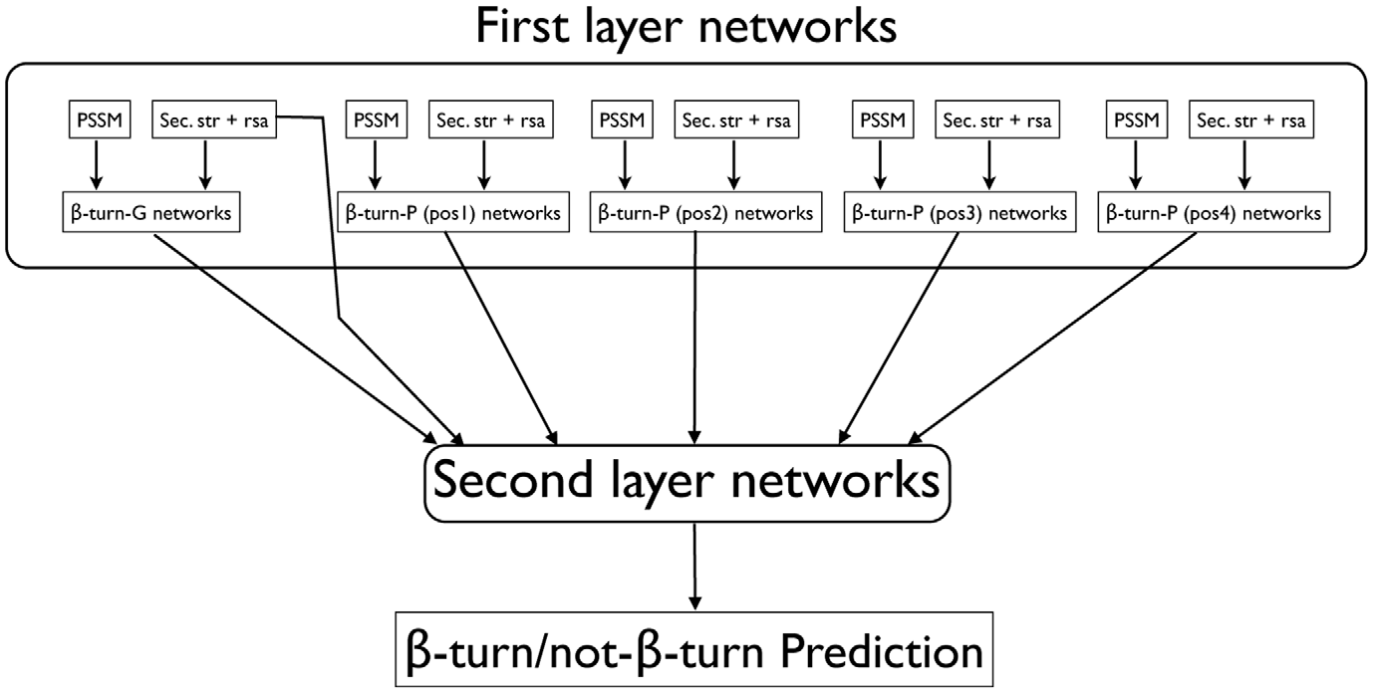
Graphical overview of the method used in training of the first and second layer networks. ‘PSSM’ is a Position-Specific Scoring Matrix. ‘Sec. str + rsa’ is secondary structure and surface accessibility predictions obtained from NetSurfP [28]. Networks with the abbreviation ‘pos’ refer to networks that predict specific positions in a β-turn. First layer networks are all ensembles of artificial neural networks where output was used for training in the second layer networks.

**Figure 2:**
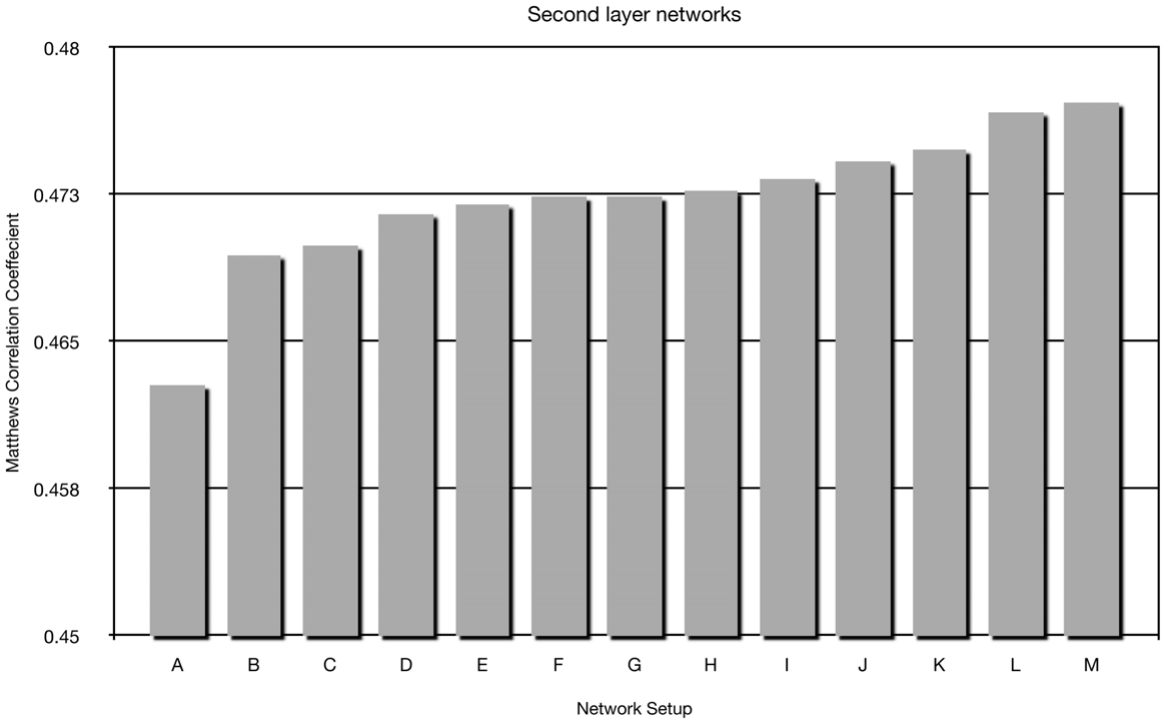
Test MCC performance on the Cull-2220 dataset, for different setups of the second level network. The performance is the average from an ensemble of 10 network architectures for each setup. Abbreviations for the setups are as follows: β-turn-P  =  position specific first layer predictions, β-turn-G  =  general β-turn/not-β-turn first layer predictions, sec-rsa  =  secondary structure and surface accessibility predictions from NetSurfP [28], PSSM  =  Position Specific Scoring Matrices. The setups are composed as follows: A =  PSSM + sec-rsa, B = PSSM + β-turn-G + sec-rsa, C =  PSSM + β-turn-G, D =  PSSM + β-turn-P, E =  β-turn-P, F =  β-turn-G + sec-rsa, G =  β-turn-G, H =  PSSM + β-turn-P + sec-rsa, I =  β-turn-P + sec-rsa, J =  PSSM + β-turn-P + β-turn-G + sec-rsa, K =  PSSM + β-turn-P + β-turn-G, L =  β-turn-P + β-turn-G, M =  β-turn-P + β-turn-G + sec-rsa.

A schematic overview of the final NetTurnP method is shown in Figure 1. The method consists of two artificial neural network layers. Several second layer network setups were tested in order to find the architecture with the highest cross-validated MCC value based on training set sequences. These different setups can be seen schematically in Table S1. The setups gave similar performances as seen in Figure 2, however, we chose the best setup (M) for the final NetTurnP method.

### First layer networks

Classification artificial neural networks, β-turn-G, were trained to predict whether or not an amino acid was located in a β-turn. Input to the networks was sequence profiles in form of PSSM's, predicted secondary structure and surface accessibility. Using 10-fold cross validation spanning a series of different network architectures, an ensemble was constructed of the best 100 network architectures, determined by cross validation leave-out tests (see methods). A cross-validated test performance of Q_total_ = 77.8%, PPV  = 51.3%, Sens  = 73.1%, MCC  = 0.47 and an AUC of 0.846 was obtained.

Furthermore, position specific networks, β-turn-P as described in materials and methods, were also trained in order to increase the predictive performance of the second level networks. Test performances for these networks can be seen in Table S2.

### Second layer networks

The output from the first layer networks was used as an input to the second layer networks. The final method uses predictions from the β-turn-P and β-turn-G networks, including secondary structure and relative surface accessibility predictions from NetSurfP [28]. An ensemble of 10 network architectures was selected corresponding to the top ranking network architecture within each of the subsets, based on the leave-out performance. Further increasing the number of architectures in the ensemble did not increase the performance (Figure S1). A cross-validated test performance of Q_total_  = 78.8%, PPV  = 53.0%, sensitivity  = 71.5% and an MCC of 0.48 with an AUC of 0.849 was obtained. Results for both the first and second layer network test performances are shown in Table S3. All performances increased from the first to the second layer networks, except for the sensitivity, which decreased 1.6 percentage points.

**Figure 3:**
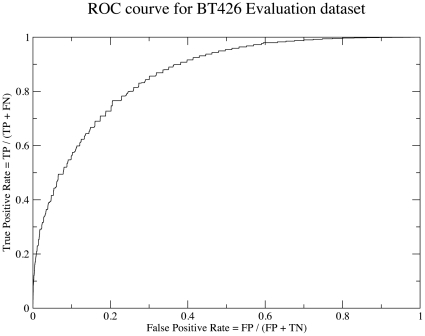
ROC curve for the evaluation of NetTurnP. The figure shows the ROC curve (True positive rate vs. False Positive Rate) for the evaluation of the NetTurnP against the BT426 dataset.

The neural network ensemble was also evaluated against the BT426 dataset. The performance values achieved were: Q_total_ = 78.2%, PPV  = 54.4%, sensitivity  = 75.6% and a MCC of 0.50 with an AUC of 0.864. The ROC curve for the evaluation of the NetTurnP is shown in Figure 3. A 7-fold cross validation performed on the BT426 dataset showed that the result obtained is very comparable to the general NetTurnP method as can be seen in Table 1.

**Table 1:** Comparison of NetTurnP with other β-turn prediction methods.

Prediction method	Q_total_	PPV	Sens	Spec	MCC	AUC
NetTurnP	78.2	54.4	75.6	79.1	0.50	0.864
NetTurnP-tweak	82.1	68.8	50.9	92.4	0.48	0.864
NetTurnP BT426 7-fold	78.1	54.4	74.2	79.5	0.49	0.853
DEBT	79.2	54.8	70.1	N/A	0.48	0.84
E-SSpred	80.9	63.6	49.2	N/A	0.44	0.84
BTNpred	80.9	62.7	55.6	N/A	0.47	N/A
SVM	79.8	55.6	68.9	N/A	0.47	0.87
MOLEBRNN	77.9	53.9	66.0	N/A	0.45	0.832
BTSVM	78.7	56.0	62.0	N/A	0.45	N/A
BetaTPred2	75.5	49.8	72.3	N/A	0.43	0.77
COUDES	75.5	49.8	66.6	N/A	0.41	N/A
KNN	75.0	46.5	66.7	N/A	0.40	N/A
BTPRED	74.9	55.3	48.0	N/A	0.35	N/A
1–4 and 2–3 correlation model	59.1	32.4	61.9	N/A	0.17	N/A

Results are based on the BT426 evaluation dataset. All performance measures have been described in the methods section. NetTurnP is referring to the final performance after the second layer networks, NetTurnP-tweak is the approach that was tweaked for best Q_total_ performance. NetTurnP BT426 7-fold is referring to a 7-fold cross-validation performed on the BT426 dataset. The other methods are as follows: DEBT [45], E-SSpred [19], BTNpred [21], SVM [22], MOLEBRNN [46], BTSVM [47], BetaTPred2 [17], COUDES [6], KNN [14], BTPRED [13] and 1–4 and 2–3 correlation model [11].

**Figure 4:**
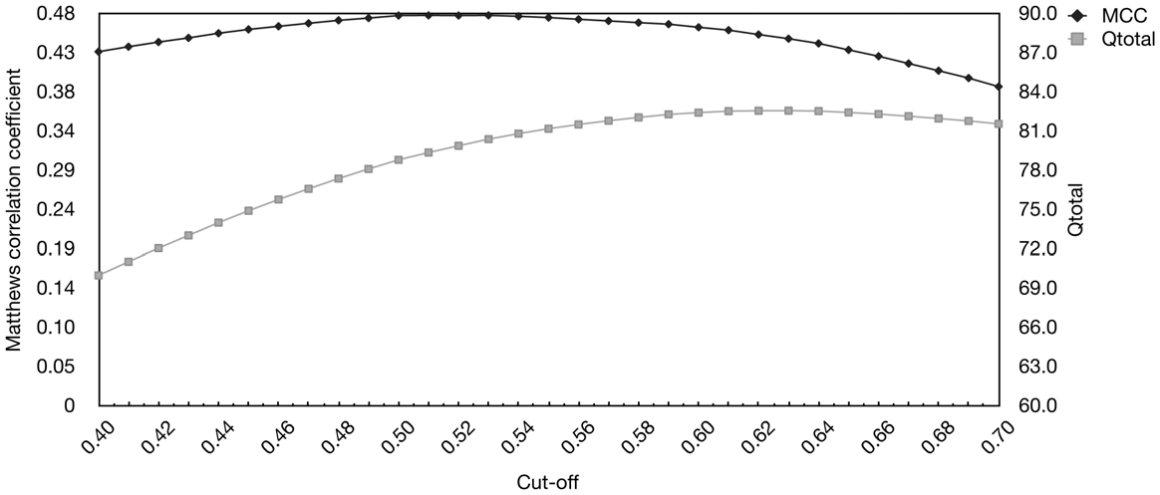
MCC and Q_total_ as function of the cut-off value. The figure shows MCC and Q_total_ as function of the cut-off value. The values are obtained by cross-validation of the Cull-2220 dataset. The X-axis is the threshold for a positive prediction of a β-turn. Y-axis to the left is the Matthews correlation coefficient and to the right Q_total_ values.

The Q_total_ measure can be optimized, but at the expense of a lower MCC and sensitivity. We analyzed this relationship by varying the cut-off for a positive prediction as seen in Figure 4. A cut-off of 0.61 gave the highest Q_total_ of 82.5% and MCC of 0.46 on the test set, whereas using our default cut-off (0.50) gave a Q_total_ of 78.8 and an MCC of 0.48.

Using this cut-off of 0.61 for the evaluation dataset resulted in a Q_total_ of 82.1% and an MCC of 0.48 as shown in Table 1.

**Figure 5:**
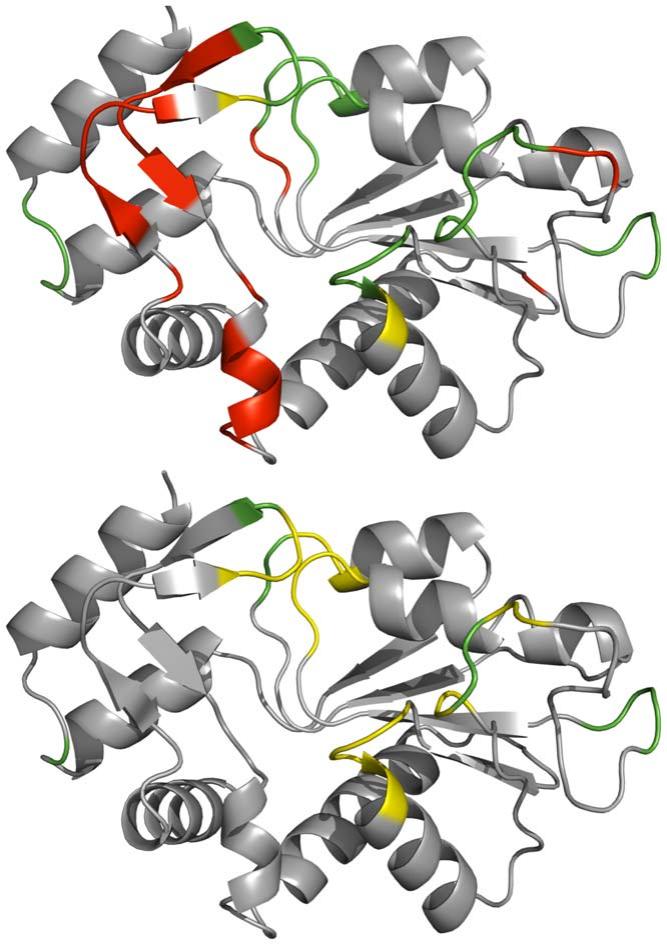
1D projection of β-turn predictions for default and Q_total_ optimized cut-off plotted on 3D structure 2WNS chain A. The figure shows the structure of a transferase, 2WNS chain A. The top structure shows a prediction where default cut-off has been used (NetTurnP) and the bottom structure shows the same structure where cut-off tweak has been applied (NetTurnP-tweak). Assigned β-turns are yellow, false positives are red, and the residues in green are where assignments and predictions agree. Figures were made using the PYMOL software [44].

Predicted and assigned β-turns are illustrated on the PDB structure 2WNS:A in Figure 5. It is a transferase with 197 amino acids where 31 amino acids were assigned by PROMOTIF as being located in a β-turn. Prediction of β-turns was done using the NetTurnP and NetTurnP-tweak methods to show the effect of a tweaked Q_total_ performance. The performance using NetTurnP on 2WNS:A gave Q_total_ = 87.3%, PPV  = 55.8%, sensitivity  = 93.6% with a MCC of 0.66 and an AUC of 0.955. Using NetTurnP-tweak the protein chain was predicted to a precision of Q_total_ = 89.3%, PPV  = 100%, sensitivity  = 32.3% with a MCC of 0.54. The AUC value was unchanged.

### β-turn-S networks

Classification networks were trained to predict whether an amino acid was located in one of the nine types of β-turn, as earlier defined. Networks were trained using the same method as described for β-turn-G i.e. an ensemble of 100 networks architectures for the first layer and 10 architectures for the second layer networks. Evaluation performances for the second layer β-turn-S networks are summarized in Table 2, along with a comparison against four other methods.

**Table 2:** Comparison of NetTurnP and other β-turn methods for prediction of specific β-turn types.

β-turn type	Method				
	MOLEBRNN	COUDES	BETATURNS	DEBT	NetTurnP
Type I	0.317	0.309	0.29	**0.36**	**0.36**
Type I'	**0.356**	0.226	N/A	N/A	0.23
Type II	**0.339**	0.302	0.29	0.29	0.31
Type II'	0.137	0.106	N/A	N/A	**0.16**
Type IV	0.236	0.109	0.23	**0.27**	**0.27**
Type VIII	0.109	0.071	0.02	0.14	**0.16**

The table shows a comparison of NetTurnP with other methods for prediction of β-turn types using the BT426 dataset. Performance values are given as Matthews correlation coefficients and the best are highlighted in bold. The methods are: MOLEBRNN [46], COUDES [6], BETATURNS [16] and DEBT [45] have all used seven-fold cross validation. We choose to completely exclude those data from the NetTurnP test and training and thus report evaluation performances against the BT426 dataset. The β-turn types VIII, V1a1 and VIa2 can only be predicted with correlations coefficients below or close to 0.1.

### Evaluation of NetTurnP method against PLP datasets

Sequences for each of the three datasets PLP399, PLP364 and PLP273 were submitted to the NetTurnP, NetTurnP-tweak and the BetaTPred2 webservers. Evaluation performances are summarized in Table 3.

**Table 3:** Evaluation of β-turn prediction on new PLP datasets.

Prediction Method	Dataset	Q_total_	PPV	Sens	Spec	MCC	AUC
NetTurnP	PLP399	78.73	52.16	69.82	81.33	0.47	0.845
	PLP364	78.83	52.07	70.23	81.32	0.47	0.847
	PLP273	78.95	51.91	70.03	81.49	0.47	0.846
NetTurnP-tweak	PLP399	82.59	67.10	44.86	93.59	0.45	0.845
	PLP364	82.66	66.67	45.32	93.45	0.45	0.847
	PLP273	82.74	66.40	45.04	93.50	0.45	0.846
Betatpred2	PLP399	74.90	45.91	62.98	78.37	0.37	N/A
	PLP364	75.01	45.84	63.20	78.42	0.38	N/A
	PLP273	75.17	45.67	62.52	78.78	0.37	N/A

The table shows a comparison of NetTurnP, NetTurnP-tweak and the Betatpred2 method [17]. The datasets PLP364 and PLP273 are subsets of PLP399, where PLP364 contain sequences deposited in PDB from 2008–2010 and PLP273 only contain sequences deposited from 2009–2010.

Evaluating NetTurnP and NetTurnP-tweak showed that both methods are very stable over all three datasets, with only 0.22% difference in Q_total_ for NetTurnP, and 0.15% for NetTurnP-tweak within the datasets. The same trend of stable prediction is seen for all other performance measures as well. NetTurnP and NetTurnP-tweak have a small decrease of 0.03 in MCC compared to the performance against BT426 (Table 1) whereas BetaTPred2 has an even bigger decrease of 0.06 in MCC. This indicates that both of the NetTurnP methods are still better than the BetaTPred2 method and now by an even bigger margin. Also, the slightly reduced MCC values indicate that the new PLP datasets contain more difficult targets compared to the original BT426 dataset,

## Discussion

In the work presented in this paper a neural-network method called NetTurnP was developed. It predicts β-turns in general and the specific type of β-turn. This work represents one of the few studies where an independent evaluation dataset was used in addition to cross-validation. The evaluation set was non-homologous to the training datasets used. NetTurnP reached a Q_total_ of 78.2% with a MCC of 0.50, using a two-layered network structure, where the predictions from the first layer networks were used as input for the second layer.

β-turns tend to be located at solvent-exposed surfaces. Analyzing our training dataset (Cull-2220), we found that the most frequently observed amino acids in β-turns compared to the amino acid at any position were: Gly (11.6%/7.2%), Asp (8.9%/5.9%), Ser (7.1%/6.1%), Pro (7.0%/4.6%), Ala (6.4%/7.8%), Asn (6.3%/4.2%) and Glu (6.3%/7.0%). These amino acid residues are hydrophilic or small, where Pro is special due to its fixed and rigid structure making it suitable to reverse the direction of a protein chain. It is seen that Gly, Asp, Ser, Pro and Asn are occurring more often in β-turns than in general, and that Ala and Glu occur less frequently. A complete table of the frequencies for all amino acids is shown in Table S4.

For the second layer networks different setups were tested in order to find the highest test (MCC) performance. We found that it was most optimal to use predictions from the networks β-turn-G and β-turn-P and with inclusion of predicted secondary structure and relative surface accessibility predictions.

The second layer networks were found to filter out the noise and increase the AUC value from 0.846 to 0.849 in test performance. This increase was found to be a significant increase corresponding to a p-value << 0.001, using an unpaired test with two independent samples [29]. For the evaluation dataset BT426 the AUC increased from 0.860 to 0.864 after primary and second level networks, respectively. (p-value << 0.001).

**Figure 6:**
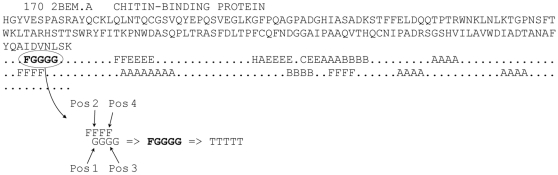
Assignment scheme used to train the β-turn-P method. Figure 6 is illustrating the assignment scheme used to train the β-turn-P method for an example protein sequence with PDB-identifier 2BEM.A. A β-turn with a length of five shown as T's, is composed of two overlapping β-turn types, here indicated with F (Type VIII) and G (Type VIa2). In this situation, one β-turn residue can be assigned as being both at position 1 and at position 2. Another β-turn residue can be assigned as being both at position 3 and at position 4.

Because of the unbalanced dataset (25% β-turns), Q_total_ is a poor measure by itself, as it is possible to achieve a Q_total_ of 75% if all residues were predicted to be non-β-turns. Instead, NetTurnP was trained to achieve the best MCC, which will also balance the performance measured on sensitivity and specificity. The effect of a tweaked Qtotal performance (NetTurnP-tweak) showed that we could obtain a better Q_total_ than any other method, but at the expense that more false and true positives are removed as seen in Table 1 and Figure 6. Therefore only the most confident predictions remain, but the method becomes less sensitive. NetTurnP, with tweaking Q_total_, achieves the best MCC performance of 0.48 compared to other methods.

For the prediction of specific β-turn types NetTurnP showed improved performance for four out of six β-turn types compared to other methods as seen in Table 2. We do provide a prediction via the webserver for the β-turn types VIb, VIa1 and Via2, even though the performances are quite low with MCC values of 0.11, 0.07 and 0.03 respectively. It is most likely due to the scarce number of these β-turn types.

Three new datasets were created with the purpose of evaluating NetTurnP and NetTurnP-tweak against a more recent set of sequences than the original dataset BT426. For the comparison NetTurnP/NetTurnP-tweak, DEBT, MOLEBRNN and BetaTPred2 were chosen. Due to errors in the DEBT and MOLEBRNN webservers, we were not able to obtain enough results for a comparison. MOLEBRNN never completed any calculations, and DEBT only succeeded to return a few results. All sequences were successfully submitted to NetTurnP/NetTurnP-tweak and BetaTpred2. Multiple sequences can be submitted to the NetTurnP webserver, which is a functionality that none of the other webservers provide.

For NetTurnP/NetTurnP-tweak the performance drops by 0.03 in terms of MCC compared to the performance obtained using the BT426 dataset. BetaTPred2 had an even bigger decrease in MCC of 0.06. This could indicate that the newer sequence data is a more challenging dataset.

A dataset of 75 experimentally determined antigen-antibody structures with predicted epitope residues was downloaded from the supplementary section of DiscoTope [30] in order to analyze the frequency of β-turns in discontinuous B-cell epitopes. We find that there is an overrepresentation with a factor 2 (data not shown) of β-turns in the discontinuous B-cell epitopes. We therefore believe that prediction of β-turns in general, can further improve immunological feature predictions.

## Materials and Methods

### Evaluation dataset, BT426

To evaluate the NetTurnP method, a dataset of 426 non-homologous protein chains was used. The dataset, commonly known as BT426, was created by Guruprasad and Rajkumar [31] and consists of >94,800 amino acids. Several groups use it as a golden set of sequences upon which performance values are reported and compared. The dataset consists of protein chains whose structure has been determined by X-ray crystallography at a resolution of 2.0 Å or better. Each chain contains at least one β-turn region. In total 23,580 amino acids, corresponding to 24.9% of all amino acids, have been assigned to be located in β-turns. None of the sequences in the dataset share more than 25% sequence identity. The BT426 dataset was downloaded from the Raghava Group's website: http://www.imtech.res.in/raghava/bteval/dataset.html. Four sequences are obsolete from PDB and superceded by newer sequence data. Therefore 1GDO.A was replaced by 1XFF.A, 5ICB by 1IG5, 1ALO by 1VLB and 3B5C by 1CYO. This dataset was solely used for the final evaluation of our NetTurnP method.

### Evaluation datasets, PLP399, PLP364 and PLP273

Three new datasets were constructed with the purpose of evaluating the NetTurnP method against a more recent set of protein sequences. Protein sequence data was extracted from the RCSB (Research Collaboratory for Structural Bioinformatics) Protein Data Bank (PDB) [32] using the protein culling server PISCES [33]. An initial dataset was created using the following criteria: Maximum sequence identify < = 25%, Resolution < = 2.0 Å, R-factor < = 0.2, sequence in the range 25 -10,000 amino acids and including X-structures only. The resulting dataset contained 3,572 PDB protein chains before homology reduction. A Hobohm1 algorithm with a threshold as described previously [34] was used to create the final homology reduced dataset PLP399, containing 399 protein chains. No sequences in the dataset share more than 25% sequence identity to a sequence within the BT426 dataset, Cull-2220 dataset (described below) or the datasets used for training and evaluation of the NetSurfP method [28]. The PLP399 dataset was further subdivided into PLP364 containing only sequences with deposition date from 2008 and newer and PLP273 containing sequences from 2009–2010. All three datasets are solely used to evaluate the NetTurnP method, and they are available for download at http://www.cbs.dtu.dk/services/NetTurnP/suppl/plp.php.

### Training dataset, Cull-2220

Protein sequence data was obtained from PDB using PISCES. A dataset was constructed in two steps, first an initial selection of potential sequences and later a more strict selection based on a Hobohm1 [35] homology reduction algorithm. First PDB was culled using the following criteria: Maximum sequence percentage identity < = 40%, Resolution < = 3.0 Å, R-factor < = 0.2, sequence length in the range 40–10,000 amino acids and including X-ray structures only. The resulting dataset contained 5,648 PDB protein chains before homology reduction. An empiric sorting function (1) was applied to rank the protein chains such that high-resolution structures with the most experimentally determined amino acids were preferred instead of the shorter low-resolution homologous protein sequence. A Hobohm1 algorithm with a threshold as described previously [34] was used to create the final homology reduced dataset (Cull-2220). No sequences in the dataset share more than 25% sequence identity to a sequence within the BT426 dataset.
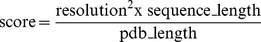
(1)


Equation 1 – Ranking of experimentally determined protein sequences. The best rank is assigned to the protein sequence with the lowest score. “resolution” is the resolution in Ångstroms according to PDB, “sequence_length” is referring to the actual length of the sequence which may include amino acids for which there are no available coordinates in the PDB-file. “pdb_length” is the length of the sequence for which there are coordinates for the amino acids.

### β-turn assignment

The program PROMOTIF [36] was used for assignment of β-turns and for the Cull-2200 dataset where 98,624 out of 451,812 amino acids were assigned to be inside a β-turn (21.8%) region.

According to restraints on phi(Φ) and psi(ψ) dihedral angles between residues *i+1* and *i+2*, nine β-turn type specific datasets were created. The Φ, ψ restrains for each of the types (I, I', II, II', IV, VIII, VIb, VIa1 and VIab) are shown in Table S5. These angles are allowed to deviate ±30° from the defined angles, with the addition that one dihedral angle is allowed to deviate as much as ±40°. Type VIa1 and VIa2 also require a cis-proline at position *i+2*.

For the general prediction of β-turns, the positive set includes the amino acid residues that belong to any of the 9 β-turn types and the negative set include all other residues. For the type specific β-turn predictions, the positive sets were reduced to include only β-turns of one specific type whereas everything else comprised a negative dataset. The number and percentage of amino acids (positive sets) in each of the type specific datasets are: type I 40,482/9.0%, type I' 4,812/1.1%, type II 14,375/3.2%, type II' 3,124/0.7%, type IV 38,445/8.5%, type VIII 11,192/2.5%, type VIb 1,120/0.3%, type VIa1 736/0.2% and type VIa2 214/0.1%.

### Position Specific Scoring Matrices

Sequence profiles i.e. Position-Specific Scoring Matrices (PSSM) were generated for all protein chains, using the iterative PsiBLAST program [37]. Query sequences were blasted for four iterations against a local copy of the National Center for Biotechnology Information (NCBI) non-redundant (nr) sequence database, which for speed purposes had been homology-reduced using CDHIT [38] to less than 70% sequence identity. An E-value cut-off of 1×10-5 was used.

### Secondary structure and surface accessibility

Secondary structure and surface accessibility predictions were generated for all protein chains, using the NetSurfP program [28].

### Neural Networks

A standard feed-forward procedure was utilized to train the neural networks [39], and a gradient descent method was used to back-propagate the errors where-after weights were updated [40]. A sliding window of amino acids was presented to the neural network and predictions were made for the central position. The neural networks were trained using window sizes of 5, 7, 9, 11 and 13, the following number of hidden units: 50, 75, 100 and 125, and two output neurons. Altogether we used 20 different neural network architectures. A 10-fold cross-validation procedure was used, thus a total of 200 neural networks. Synapse weights were stored for the epoch where the best test set Matthews correlation coefficient was obtained.

Amino acids were encoded both using PSSM values, three neurons for predicted helix, strand and coil and one extra neuron for the relative surface accessibility, thus a total of 25 neurons were used to describe an amino acid.

### Optimized Networks

Three different types of artificial neural networks have been trained:

- β-turn-G
- β-turn-S
- β-turn-P

The β-turn-G (G for general) method predicts if an amino acid is located in a β-turn region or not.

The β-turn-S (S for specific) method was trained to predict if an amino acid belongs to any of the nine β-turn classes: I, I', II, II', IV, VIII, VIb, VIa1 and VIab.

The method β-turn-P (P for position) is a combination of four sub-methods that have been trained to predict if an amino acid is located at position 1, position 2, position 3 or position 4 in a β-turn. Some amino acids can be assigned to multiple positions within a β-turn as shown in Figure 1. However, within each of the four sub-methods only one position was considered.

We found that the performance of the methods β*-turn-G* and β*-turn-S* could be improved by use of a second layer of neural networks where information from the β*-turn-P* method was included as input. A second layer is often used as some of false predictions can be corrected [28], [41] and is due to the fact that new or enriched input data is provided for the second layer neural networks.

### Performance measures

The quality of the predictions was evaluated using six measures; Matthews correlation coefficient [42] (MCC), Q_Total_, Predicted Positive Value (PPV), sensitivity, specificity and Area under the Receiver Operating Curve [43] (AUC). FP  =  False Positive, FN  =  False Negative, TP  =  True Positive, TN  =  True Negative.
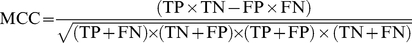
(2)


Matthews correlation coefficient can be in the range of −1 to 1, where 1 is a perfect correlation and -1 is the perfect anti-correlation. A value of 0 indicates no correlation. 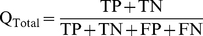
(3)


Q_total_ is the percentage of correctly classified residues, also called the prediction accuracy. 
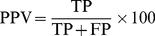
(4)


PPV is the Predicted Positive Value, also called the precision or Q_pred_. 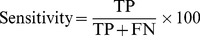
(5)


Sensitivity is also called recall or Q_Obs_, and is the fraction of the total positive examples that are correctly predicted.
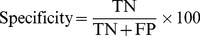
(6)


Specificity is the fraction of total negative examples that are correctly predicted.

The above-mentioned performance measures are all threshold dependent and in this work a threshold of 0.5 was used, unless otherwise stated.

AUC is a threshold independent measure, and was calculated from the ROC curve which is a plot of the sensitivity against the False Positive rate  =  FP/(FP + TN). An AUC value above 0.7 is an indication of a useful prediction and a good prediction method achieves a value >0.85 [40].

## Supporting Information

Table S1 **setups tested for training in the second layer networks.**
The table is listing the different setups tested for training in the second layer networks. In the table abbreviations are as follows: β-turn-G  =  β-turn/not-β-turn prediction from first layer networks, β-turn-P =  position specific predictions from first layer networks, sec-rsa  =  secondary structure and surface accessibility predictions from NetSurfP [28], PSSM  =  Position Specific Scoring Matrices.
(DOCX)

Table S2 **test performance for the first layer β-turn-P networks.**
Test performances from the first layer β-turn-P networks using the Cull-2220 dataset. All performance measures have been explained in the methods section. All β-turn-P networks were trained using pssm + sec + rsa, where pssm  =  Position Specific Scoring Matrix, sec  =  Secondary structure predictions [28], rsa  =  Relative solvent accessibility predictions [28]. The positions in the four network trainings are referring to the position in a β-turn.
(DOCX)

Table S3 **Test performances from the first and second layer β-turn-G networks using the Cull-2220 dataset.** All performance measures have been explained in the methods section. The first layer networks were using pssm + sec + rsa, and the secondary networks were using β-turn-P + β-turn-G + sec + rsa, where the used nomenclature are: pssm  =  Position Specific Scoring Matrix, sec  =  secondary structure predictions [28], rsa  =  relative solvent accessibility predictions [28]. β-turn-G  =  β-turn/non-β-turn predictions, β-turn-P  =  predictions from the position specific networks.
(DOCX)

Table S4 **Amino acid statistics in Cull-2200 dataset.**
Frequencies for amino acids in β-turns and the Cull-2220 training set. The first part of the table ‘β-turn statistics’ shows the amount of residues, which have been assigned as β-turns and their percentage of the total amount of β-turn assigned residues in the Cull-2220 set. The second part of the table ‘Amino acid statistics’ shows the amount of residues and the percentage of the total Cull-2220 set.
(DOCX)

Table S5 **Dihedral angles for the β-turn types as used by PROMOTIF**
Dihedral angles for the β-turn types between residues two (*i+1*) and three (*i+2*) as used by PROMOTIF [39]. These angles are allowed to deviate by ±30° from the defined angles, with the addition that one dihedral angle is allowed to deviate as much as ±40°. Type IV is used for all β-turns, which do not fall within the dihedral angle ranges for the eight defined types. Type VIa1, VIa2 also require a cis-proline at position *i+2*.
(DOCX)

Figure S1 **Matthews correlation using different setups and an increasing number of trained network architectures.**
The figure shows test performances in Matthews's correlation coefficient when including an increasing number of trained networks architectures, named *Top ranked network architectures*, based on test set performance using different setups. Abbreviations for the setups are as follows: β-turn-P  =  position specific first layer predictions, β-turn-G  =  general β-turn/not-β-turn first layer predictions, sec-rsa  =  secondary structure and surface accessibility predictions from NetSurfP [28], PSSM  =  Position Specific Scoring Matrices. The setups are composed as follows: A =  PSSM + sec-rsa, B = PSSM + β-turn-G+ sec-rsa, C =  PSSM + β-turn-G, D =  PSSM + β-turn-P, E =  β-turn-P, F =  β-turn-G + sec-rsa, G =  β-turn-G, H =  PSSM + β-turn-P + sec-rsa, I  =  β-turn-P + sec-rsa, J  =  PSSM + β-turn-P + β-turn-G + sec-rsa, K =  PSSM + β-turn-P + β-turn-G, L =  β-turn-P + β-turn-G, M =  β-turn-P + β-turn-G + sec-rsa.
(TIFF)
