# Supplementary material for: NetTurnP – Neural Network Prediction of Beta-turns by Use of Evolutionary Information and Predicted Protein Sequence Features
Source: PLoS One. 2010 Nov 30;5(11):e15079. doi: 10.1371/journal.pone.0015079 (PMC2994801; doi:10.1371/journal.pone.0015079)
Supplement: Table S1 — setups tested for training in the second layer networks. The table is listing the different setups tested for training in the second layer networks. In the table abbreviations are as follows: β-turn-G = β-turn/not-β-turn prediction from first layer networks, β-turn-P = position specific predictions from first layer networks, sec-rsa = secondary structure and surface accessibility predictions from NetSurfP [28], PSSM = Position Specific Scoring Matrices. (DOCX) [file pone.0015079.s001.docx]

**Table S1 – setups tested for training in the second layer networks.**

| Setup | pssm | sec-rsa | β-turn-G | β-turn-P |
| --- | --- | --- | --- | --- |
| A | X | X |  |  |
| B | X | X | X |  |
| C | X |  | X |  |
| D | X |  |  | X |
| E |  |  |  | X |
| F |  | X | X |  |
| G |  |  | X |  |
| H | X | X |  | X |
| I |  | X |  | X |
| J | X | X | X | X |
| K | X |  | X | X |
| L |  |  | X | X |
| M |  | X | X | X |
