# Supplementary material for: NetTurnP – Neural Network Prediction of Beta-turns by Use of Evolutionary Information and Predicted Protein Sequence Features
Source: PLoS One. 2010 Nov 30;5(11):e15079. doi: 10.1371/journal.pone.0015079 (PMC2994801; doi:10.1371/journal.pone.0015079)
Supplement: Table S2 — test performance for the first layer β-turn-P networks. Test performances from the first layer β-turn-P networks using the Cull-2220 dataset. All performance measures have been explained in the methods section. All β-turn-P networks were trained using pssm + sec + rsa, where pssm = Position Specific Scoring Matrix, sec = Secondary structure predictions [28], rsa = Relative solvent accessibility predictions [28]. The positions in the four network trainings are referring to the position in a β-turn. (DOCX) [file pone.0015079.s002.docx]

**Table S2 – test performance for the first layer β-turn-P networks.**

| **β-turn-P networks** | **Q_total_** | **PPV** | **Sens** | **Spec** | **MCC** | **AUC** |
| --- | --- | --- | --- | --- | --- | --- |
| β-turn-P (Position 1) | 84.0 | 26.1 | 65.9 | 85.4 | 0.34 | 0.849 |
| β-turn-P (Position 2) | 83.5 | 25.7 | 67.4 | 84.8 | 0.34 | 0.852 |
| β-turn-P (Position 3) | 83.5 | 25.7 | 67.2 | 84.8 | 0.34 | 0.852 |
| β-turn-P (Position 4) | 83.5 | 25.6 | 67.0 | 84.8 | 0.34 | 0.851 |
