# Supplementary material for: NetTurnP – Neural Network Prediction of Beta-turns by Use of Evolutionary Information and Predicted Protein Sequence Features
Source: PLoS One. 2010 Nov 30;5(11):e15079. doi: 10.1371/journal.pone.0015079 (PMC2994801; doi:10.1371/journal.pone.0015079)
Supplement: Table S3 — Test performances from the first and second layer β-turn-G networks using the Cull-2220 dataset. All performance measures have been explained in the methods section. The first layer networks were using pssm + sec + rsa, and the secondary networks were using β-turn-P + β-turn-G + sec + rsa, where the used nomenclature are: pssm = Position Specific Scoring Matrix, sec = secondary structure predictions [28], rsa = relative solvent accessibility predictions [28]. β-turn-G = β-turn/non-β-turn predictions, β-turn-P = predictions from the position specific networks. (DOCX) [file pone.0015079.s003.docx]

**Table S3 - test performances from the first and second layer β-turn-G networks.**

| **Network level** | **Q_total_** | **PPV** | **Sens** | **Spec** | **MCC** | **AUC** |
| --- | --- | --- | --- | --- | --- | --- |
| First layer networks | 77.8 | 51.3 | 73.1 | 79.1 | 0.47 | 0.846 |
| Second layer networks | 78.8 | 53.0 | 71.5 | 81.0 | 0.48 | 0.849 |
