# Supplementary material for: NetTurnP – Neural Network Prediction of Beta-turns by Use of Evolutionary Information and Predicted Protein Sequence Features
Source: PLoS One. 2010 Nov 30;5(11):e15079. doi: 10.1371/journal.pone.0015079 (PMC2994801; doi:10.1371/journal.pone.0015079)
Supplement: Table S4 — Amino acid statistics in Cull-2200 dataset. Frequencies for amino acids in β-turns and the Cull-2220 training set. The first part of the table ‘β-turn statistics’ shows the amount of residues, which have been assigned as β-turns and their percentage of the total amount of β-turn assigned residues in the Cull-2220 set. The second part of the table ‘Amino acid statistics’ shows the amount of residues and the percentage of the total Cull-2220 set. (DOCX) [file pone.0015079.s004.docx]

**Table S4 - Amino acid statistics in Cull-2200 dataset.**

| **β-turn statistics** | | | | **Amino acid statistics** | |
| --- | --- | --- | --- | --- | --- |
| **Amino Acid** | | **Amount** | **% of all** | **Amount** | **% of all** |
| Ala | A | 6277 | 6.4 | 35923 | 7.95 |
| Cys | C | 1447 | 1.5 | 6081 | 1.35 |
| Asp | D | 8779 | 8.9 | 26660 | 5.90 |
| Glu | E | 6240 | 6.3 | 31514 | 6.98 |
| Phe | F | 3378 | 3.4 | 18155 | 4.02 |
| Gly | G | 11426 | 11.6 | 32443 | 7.18 |
| His | H | 2627 | 2.7 | 12206 | 2.70 |
| Ile | I | 3416 | 3.5 | 25623 | 5.67 |
| Lys | K | 5636 | 5.7 | 26234 | 5.81 |
| Leu | L | 5853 | 5.9 | 41056 | 9.09 |
| Met | M | 1107 | 1.1 | 10605 | 2.35 |
| Asn | N | 6254 | 6.3 | 19059 | 4.22 |
| Pro | P | 6865 | 7.0 | 20693 | 4.58 |
| Gln | Q | 3364 | 3.4 | 17489 | 3.87 |
| Arg | R | 4505 | 4.6 | 23249 | 5.15 |
| Ser | S | 6998 | 7.1 | 27414 | 6.07 |
| Thr | T | 5417 | 5.5 | 24113 | 5.34 |
| Val | V | 4811 | 4.9 | 31213 | 6.91 |
| Trp | W | 1224 | 1.2 | 6353 | 1.41 |
| Tyr | Y | 3018 | 3.1 | 15723 | 3.48 |
|  | | | | | |
| Total |  | 98642 | 100 | 451806 | 100 |
