# Supplementary material for: NetTurnP – Neural Network Prediction of Beta-turns by Use of Evolutionary Information and Predicted Protein Sequence Features
Source: PLoS One. 2010 Nov 30;5(11):e15079. doi: 10.1371/journal.pone.0015079 (PMC2994801; doi:10.1371/journal.pone.0015079)
Supplement: Table S5 — Dihedral angles for the β-turn types as used by PROMOTIF Dihedral angles for the β-turn types between residues two (i+1) and three (i+2) as used by PROMOTIF [39]. These angles are allowed to deviate by ±30° from the defined angles, with the addition that one dihedral angle is allowed to deviate as much as ±40°. Type IV is used for all β-turns, which do not fall within the dihedral angle ranges for the eight defined types. Type VIa1, VIa2 also require a cis-proline at position i+2. (DOCX) [file pone.0015079.s005.docx]

**Table S5 - Dihedral angles for the β-turn types as used by PROMOTIF**

| β-turn types | *Φ*, *ψ* (*i + 1*) | *Φ*, *ψ* (*i + 2*) |
| --- | --- | --- |
| I | -60°, -30° | -90°, 0° |
| I’ | 60°, 30° | 90°, 0° |
| II | -60°, 120° | 80°, 0° |
| II’ | 60°, -120° | -80°, 0° |
| VIII | -60°, -30° | -120°, 120° |
| VIa1 | -60°, 120° | -90°, 0° |
| VIa2 | -120°, 120° | -60°, 0° |
| VIba | -135°, 135° | -75°, 160° |
| IV | β-turns excluded from the above categories | |
