# Supplementary figures and images for: NetTurnP – Neural Network Prediction of Beta-turns by Use of Evolutionary Information and Predicted Protein Sequence Features
Source: PLoS One. 2010 Nov 30;5(11):e15079. doi: 10.1371/journal.pone.0015079 (PMC2994801; doi:10.1371/journal.pone.0015079)

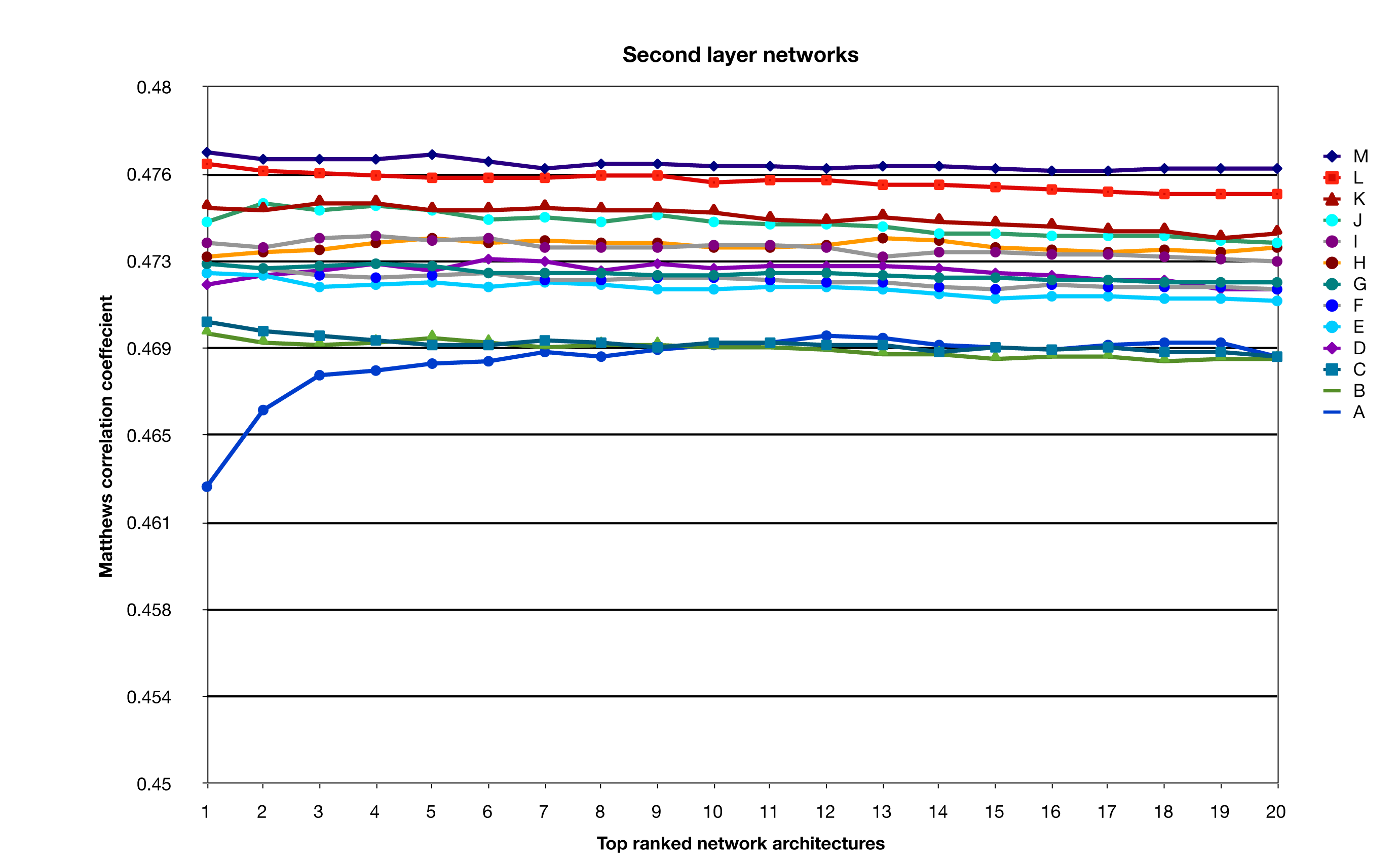

Supplement: Figure S1 — Matthews correlation using different setups and an increasing number of trained network architectures. The figure shows test performances in Matthews's correlation coefficient when including an increasing number of trained networks architectures, named Top ranked network architectures, based on test set performance using different setups. Abbreviations for the setups are as follows: β-turn-P = position specific first layer predictions, β-turn-G = general β-turn/not-β-turn first layer predictions, sec-rsa = secondary structure and surface accessibility predictions from NetSurfP [28], PSSM = Position Specific Scoring Matrices. The setups are composed as follows: A = PSSM + sec-rsa, B = PSSM + β-turn-G+ sec-rsa, C = PSSM + β-turn-G, D = PSSM + β-turn-P, E = β-turn-P, F = β-turn-G + sec-rsa, G = β-turn-G, H = PSSM + β-turn-P + sec-rsa, I = β-turn-P + sec-rsa, J = PSSM + β-turn-P + β-turn-G + sec-rsa, K = PSSM + β-turn-P + β-turn-G, L = β-turn-P + β-turn-G, M = β-turn-P + β-turn-G + sec-rsa. (TIFF) [file pone.0015079.s006.tiff]
